# Dermatologists' Way of Informative Content About Dermatology and Cosmetology on Social Media

**DOI:** 10.1111/jocd.70148

**Published:** 2025-07-21

**Authors:** Simge Ünal, Neslihan Demirel Öğüt

**Affiliations:** ^1^ Faculty of Medicine, Department of Dermatology and Venereology Uşak University, Uşak Training and Research Hospital Uşak Turkey

**Keywords:** cosmetology, dermatology, digital platforms, social media, WhatsApp

## Abstract

**Background:**

Social media (SM) plays a critical role in the dissemination of information, particularly in dermatology and cosmetology, where misinformation is prevalent. However, the spread of misinformation by non‐experts poses risks to public health. Dermatologists' participation in SM can help correct inaccuracies and improve public health knowledge. This study aims to evaluate the practices of Turkish dermatologists in creating and sharing dermatology and cosmetology‐related content on SM platforms.

**Material and Methods:**

An online survey consisting of 36 questions was distributed to Turkish dermatologists via WhatsApp, a closed Facebook group, and email. The survey gathered data on the dermatologists' demographics, SM usage, and content creation practices. The survey was conducted over a three‐month period from May to August 2024. Descriptive statistics were used for analysis.

**Results:**

Among the 137 respondents, 34% reported sharing informative content on SM, with WhatsApp and Instagram being the most used platforms. Common topics included acne (87%), cosmetic procedures like botulinum toxin (80%), and skincare advice (53%). However, only a small percentage consistently cited scientific references, and many were unaware of the legal and ethical implications of sharing patient images. The majority of dermatologists (79%) believed their posts had a positive effect on patients' decisions to use their medical services.

**Conclusion:**

While the use of social media by dermatologists to disseminate health information is growing, the quality and ethical standards of the content shared still need improvement. Dermatologists should play a more active role in creating evidence‐based, informative content, especially regarding serious dermatological conditions like skin cancer, which are less frequently covered. Additionally, there is a need for greater awareness and training on the ethical and legal aspects of using SM, particularly regarding patient privacy and informed consent. The presence of dermatologists on SM can help counteract the spread of misinformation and ensure that the public has access to accurate and reliable dermatological and cosmetic information.

## 1 Introduction

Given that about 50% of the 4.76 billion global internet users spend 2.5 h per day on digital platforms, it is reasonable to argue that social media (SM) has a significant impact on communities [1]. This impact is most noticeable in people's information‐seeking behavior on almost every topic. On SM and digital platforms, accurate and false information is disseminated in the dermatology area, just like in any other subject [2].

According to a survey, only 4% of the influencers who posted dermatological content were dermatologists, while 27% of them claimed to be skincare specialists without any credentials [3]. This raises concerns about the accuracy of the information contained in the shared content. Inaccurate information from unreliable sources puts the public's health at risk, misleads people with dermatologically useless treatment methods, and causes people to spend unnecessary money.

It is important for dermatologists to produce active content on SM and to correct common misinformation on dermatologic and cosmetologic issues. SM content produced by dermatologists should be created with information obtained from reliable sources and shared in a way that attracts the attention of society [4].

Several critical questions must be addressed: What is the prevalence of dermatologists sharing informative posts on SM? What should be the depth of dermatology and cosmetology education in society through SM? To answer these questions, we aimed to conduct an online survey to get Turkish dermatologists' opinions and attitudes on SM about creating and sharing informative content.

## 2 Material and Methods

A descriptive questionnaire consisting of 36 questions was designed by the authors using Google forms. The questionnaire is presented at Table S1. The questionnaire was shared with dermatologists through WhatsApp groups, a closed Facebook group of the Turkish Dermatology Association, and e‐mail thrice at certain time points. Informed consent was obtained online, and dermatologists who consented to participate in the survey were sent a 36‐question questionnaire. The identities of the dermatologists who participated in the survey were kept confidential. Due to the content and nature of the survey study, ethics committee approval was not required.

In the first part of the questionnaire, sociodemographic characteristics of dermatologists were obtained. The second part focused on the characteristics of social media use of dermatologists and their attitudes in creating informative content on dermatology and cosmetology.

Data were collected from May 2024 to August, 2024. Descriptive statistics were presented as numbers and percentages for questions and answers.

## 3 Result

**FIGURE 1:**
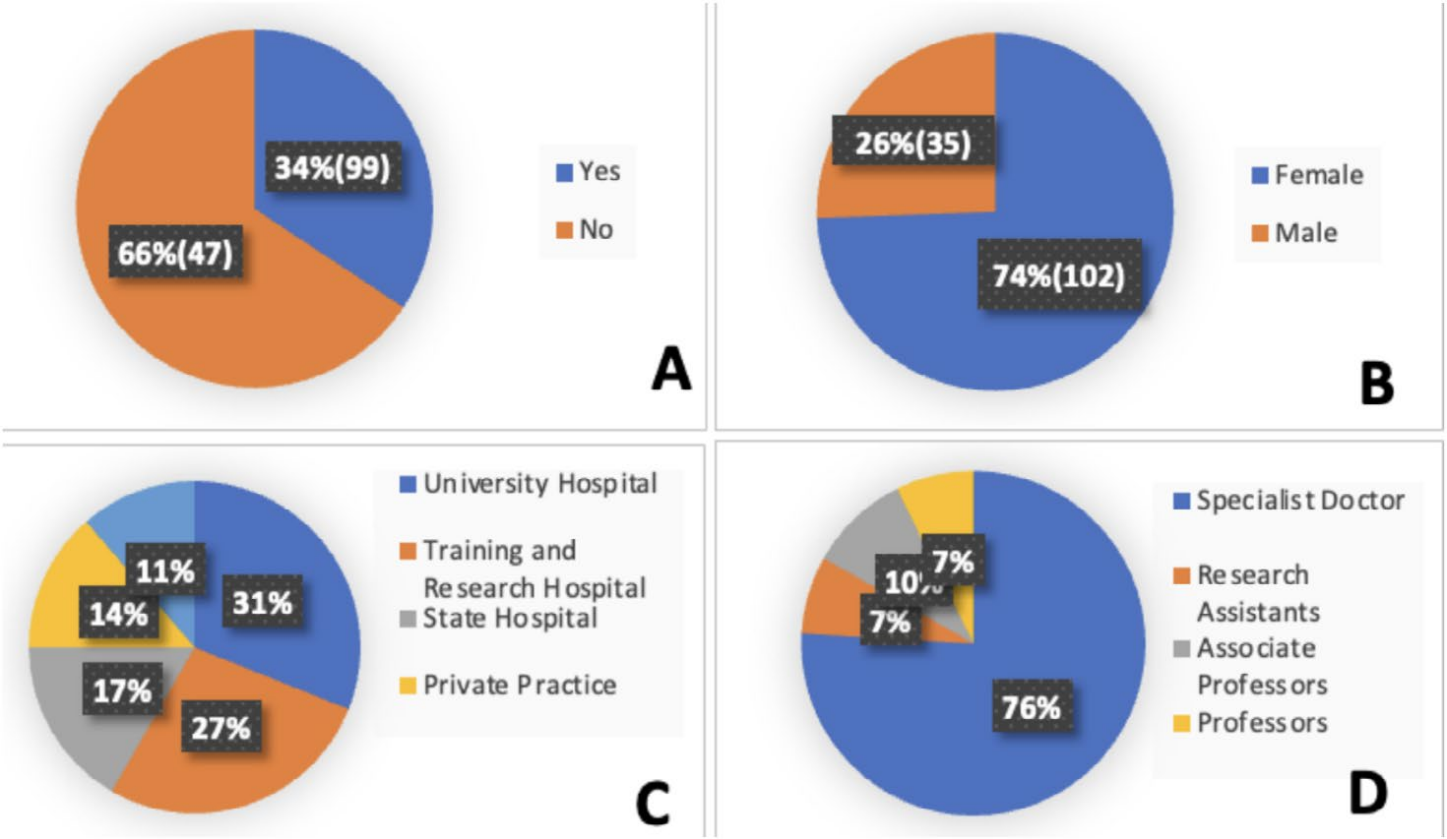
(A) Sex of the participant, (B) Title of Dermatologists (C) The institution of Dermatologists (D) Actively used social media to share information on dermatology and cosmetology.

A total of 137 dermatologists responded to the survey. Thirty‐five (26%) of them were men. The mean age of the dermatologists was 36 ± 10.3 years. The sociodemographic and occupational characteristics of dermatologists are represented in Figure 1.

### 3.1 How Do Dermatologists Use Social Media?

**FIGURE 2:**
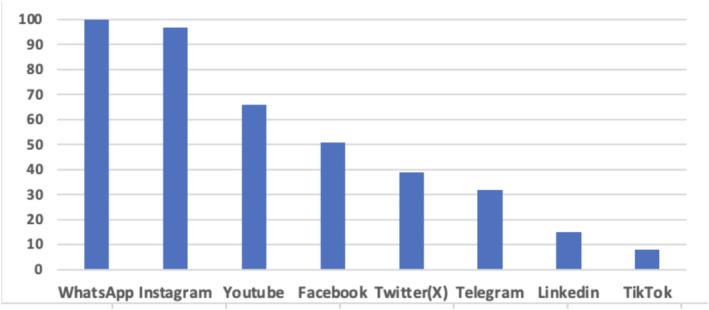
Social media application use of dermatologists (%).

All dermatologists who participated in the survey were actively using WhatsApp. The second most used SM platform was Instagram (89%). The other SM platforms used by dermatologists are represented in Figure 2. Forty‐seven (34%) of the dermatologists stated that they shared informative content about dermatology and cosmetology on their SM platforms. Only 13 dermatologists collaborated with an SM consultant to create their content. In order to increase visibility and popularity on SM, 21 dermatologists used sponsored advertisements.

Most of the active users of SM providing dermatologic information created their SM content based on diseases encountered in daily practice (78%), followed by considering the posts of other dermatologists (46%), asking followers (22%), considering influencer posts (22%), and considering the number of likes and interactions (17%).

The most common content shared by dermatologists was cosmetic procedures (78%), followed by information about daily skin/hair/nail care (53%), skin cancer and skin cancer prevention (29%), and rare diseases (17%). Acne (87%) was the most trending topic of dermatologists in terms of sharing information. This was followed by informative content about rosacea (76%), hair loss (76%), seborrheic dermatitis (70%), sun sensitivity (53%), skin cancers (48%), eczema (42%), psoriasis (42%), urticaria (44%), warts/viral diseases (36%), fungal diseases (29%), dry skin (29%), parasitic diseases (25%), and bacterial diseases (8%). Only eight dermatologists (17%) preferred to provide information on relatively rare diseases. Their preferences for sharing information about rare diseases were ichthyosis/epidermolysis bullosa, melanoma, and connective tissue disorders.

Informative SM content on cosmetic procedures shared by dermatologists was about botulinum toxin (80%), filler applications (72%), platelet‐rich plasma (PRP) and mesotherapy applications (68%), energy‐based devices (51%), thread applications (12%), and laser lipolysis (19%). Thirty‐four dermatologists (72%) stated that they recommended treatment modalities to their followers, such as home remedies, dermo‐cosmetic products, cosmetic applications, and medical treatments.

The common methods used to share informative SM content by dermatologists were video shooting (72%), article sharing (42%), patient photo/diagnosis sharing (38%), question‐answer activities (31%), and live broadcasting (4%). Forty‐two (89%) dermatologists stated that they obtained patient consent before sharing posts containing photos/videos of patients. Of these dermatologists, just one was completely non‐informed about patient privacy and the Personal Data Protection Law, whereas the other 13 were somewhat informed, 11 were very informed, and 21 were fairly informed.

Only five dermatologists stated that they shared every post with references, and 13 did not share any references at all. Literature data (93%), books (57%), information brochures of dermatology associations (42%), Google (14%), and YouTube (4%) were the most used resources for information.

Twenty‐four dermatologists (51%) preferred to answer direct questions asked individually by their followers. These individual messages were positively used by 12 dermatologists for content production by including these questions in their posts.

There were 33 dermatologists who reported that the language they used in their informational SM content was understandable to the public, 8 dermatologists used more medical terms, and 6 dermatologists used both.

### 3.2 What Do Dermatologists Think About Social Media Posts?

When asked about their motivation for using SM for informational purposes, the most common answers were to be visible (74%), to inform the public about dermatologic issues (70%), to increase the number of followers (53%), self‐promotion (42%), interaction with other dermatologists (21%), and to make money (21%).

Most of the dermatologists (79%) believe that the posts they make positively affect the patients' choice of dermatologist.

Twenty‐five dermatologists state that SM posts have a positive impact on chronic disease management. Twenty‐one dermatologists emphasize that information from SM accounts of institutions (associations etc.) may be more effective.

Twelve dermatologists state that they do not mind giving treatment recommendations. Thirty‐one dermatologists think that sharing photos/videos of cosmetic applications on SM is convenient.

## 4 Discussion

In addition to altering our conception of beauty, the rise in SM use in recent years and the filtered photos presenting flawless skin we frequently see on these platforms have inspired many people to seek the best rather than the better. Influencers have been a big part of these expectations, and people's search for unreasonable expectations has been fueled by miracle product advertising [5]. Therefore, dermatologists should use social media more frequently, offer advice based on information that is verified by science, and cite references in their posts. In this survey study, we evaluated the use of SM by dermatologists in Turkey, their purpose of using SM professionally, and their approach to informative sharing on SM as dermatologists. We expected more dermatologists to be interested in this issue and to participate in our survey. However, due to the low participation numbers of dermatologists in our study, we would like to emphasize the need for dermatologists' awareness, presence, and activism on SM.

Forty‐seven (34%) of the 137 dermatologists who participated in the survey reported using SM to create informative posts about topics related to cosmetology and dermatology. The most popular topics were acne, skin, hair, and nail care, and cosmetic applications that have gained greater attention recently. This result in our study coincides with the expectations of society from SM. In a study, it was reported that 45% of the 130 participants used SM to learn about acne treatments through YouTube (58%) and Instagram (58%) as the most popular platforms. Eighty‐one percent of them stated they took nonprescription treatments according to recommendations from these platforms. Only 31% of those treatments were convenient according to the American Academy of Dermatology's acne criteria [6]. Another survey reported that 27% of influencers who shared dermatological content in SM claimed to be skincare experts but lacked any credentials, and only 4% of them were dermatologists [3]. Inadequate controls on health‐related SM posts and the absence of scientific evidence in these posts may lead to the spread of inefficient and even dangerous content that negatively impacts public health [7]. For instance, acne treatment recommendations made by non‐dermatologists on SM may lead to an increase in the number of patients presenting with acne scars in the future and more expensive procedures to repair these scars [8, 9]. In another study analyzing YouTube videos about psoriasis, 11% of the content contained potentially harmful practices such as applying razors to psoriasis plaques, waxing, and applying vinegar [10]. Developing regulations and control mechanisms for posts on SM platforms in the field of health and dermatology would be an important step toward protecting public health. Dermatologists should address and correct false treatment suggestions and information offered by unqualified influencers on SM.

In our study, we observed that rare but important dermatological conditions such as skin cancers were less used to create informative content by dermatologists compared to cosmetic problems. According to a previous study performed in Romania, most users complied with SM instructions to prevent skin malignancies, such as avoiding sun exposure and applying SPF. Moreover, frequent SM users were more open to dermatological recommendations. It has been proposed that digital platforms could be the ideal platform for spreading awareness campaigns about skin cancer [11]. According to 44% of dermatologists in our study, information sharing through an associated institution may be more successful. Correct information about dermatological conditions can reach society by using institutional SM pages such as the Facebook page of the Turkish Society of Dermatology. From this perspective, we advise dermatologists to make educational posts from accounts of reliable dermatological associations in terms of public health as well as from their individual SM accounts.

Users can share anything they want on SM without having to provide any evidence. If these posts contain incorrect information, it can spread and easily be believed by the community. Therefore, sharing content on SM with references has acquired importance. There is increasing evidence that misinformation spreads more widely and is taken more seriously than accurate information [12]. Using anti‐vaccine propaganda as an example, there have been some unfavorable effects from the recent rise in the sharing of non‐evidence‐based anti‐vaccine content on SM. For instance, anti‐vaccine marketing has been linked to the comeback of diseases like measles, which were long since eradicated in nations like the UK, the USA, Germany, and Italy [13, 14]. Just five dermatologists in our study consistently referred to scientific sources in their posts. If health professionals make it routine to cite references in their posts, users may become accustomed to seeing references in SM posts, and posts without references may be questioned by the public and not taken at face value right away.

In our study, we found that most dermatologists who made informative posts on SM focused on cosmetic issues and the most shared topic on cosmetology was botulinum toxin applications. A study conducted by searching the hashtag “botox” on Twitter showed that most of the SM users sharing this content were physicians established in the fields of plastic surgery or dermatology [15]. Hashtags on TikTok about non‐surgical cosmetic applications were investigated in another study. Three hundred forty videos were included in the study. The majority of these videos (*n* = 126, 37.1%) and patient stories (*n* = 130, 38.2%) were provided by healthcare personnel who were not board‐certified medical doctors. In this study, posts of healthcare professionals and non‐healthcare professionals were compared regarding the median views, likes, comments, shares, and engagement. It was found that the posts of non‐healthcare influencers were significantly more popular (*p* < 0.001). Physician‐created material had considerably higher information reliability than non‐physicians' and non‐physician healthcare professionals' content (*p* < 0.001 and *p* = 0.001, respectively), according to an analysis of the content's DISCERN scores [16]. More dermatologists should be encouraged to share scientific information on cosmetic applications.

Being visible was the most frequently given response (74%), according to our survey exploring motivations of dermatologists to use SM, and most of the dermatologists (79%) believed that the posts they made positively affected the patients' choice of dermatologist. In line with our results, another research found that 87% of physicians who posted content about dermatology also shared self‐promotional content [17]. In a study, 39.7% of physicians believed that having a public SM account offered them an opportunity to promote themselves. According to the same survey, 38% of doctors believed using SM for interacting with patients was suitable from a professional perspective, 28% believed that expecting a diagnosis via SM was inappropriate, and 33.5% believed that seeking treatment via SM was inappropriate [18]. Of the dermatologists in our study, 25% stated there was no harm in recommending treatments to their followers. Additionally, half of the dermatologists used direct messaging to respond to questions from their followers. This could be due to a variety of factors, including self‐promotion and a desire to get more followers. However, in a study conducted in our country, online counseling requests from dermatologists through social media and instant messaging services were found to be associated with psychological burden and burnout [19]. Further research on this topic is required to determine ways to achieve balance between the two extremes.

Thirty‐four dermatologists (72%) in our study stated that they recommended treatment modalities to their followers through SM. Nineteen (40%) of the participants in our survey suggested dermo‐cosmetic products on social media. When we take into account social media users who are not dermatologists and even those who merely promote products because they themselves use them, this rate is incredibly low. Another topic that needs to be covered is maintaining trust while sharing and avoiding conflicts of interest with sponsored content. Avoid making bold statements like miraculous(!) products [7].

Dermatologists typically generate photo‐heavy social media posts, and for ethical reasons, it is advised not to share patient photos on SM without permission [20]. Only eleven (23%) dermatologists who answered our questions were aware of patient privacy and the Personal Data Protection Law, which could have legal consequences if someone shares information without being aware of it. The attraction of dermatologists to share information on SM has presented moral and ethical issues in recent years. In one survey, when asked if SM use by healthcare professionals complied with workplace SM policy, 31% of respondents said they were unaware of the guidelines [21]. Aside from a legal perspective, how ethical is it to share patients' photos or personal data after obtaining consent? Dermatologists can receive training or support from SM consultants to produce better‐quality content and adhere to ethical values. We suggest that dermatologists should use digital platforms more actively about their work, both individually and through associations, without deviating from ethical understandings, so that the spread of misinformation will decrease, and it will be easier for the public to access correct information.

There are limitations of our study. More dermatologists could have participated in our study. The small sample size limits the generalizability of the study. The use of digital platforms may vary between countries and cultural values. Considering the legal processes, there may be differences in sharing restrictions between countries. Although the survey questions were not validated, they were sufficient to report the current situation of dermatologists' way of using SM in Turkey.

## 5 Conclusion

In the field of dermatology, we observe that a lot of patients seek information about their conditions on digital platforms before visiting a professional. Unfortunately, incorrect information spreads more quickly than accurate information, and there are currently no guidelines for posting any kind of information on social media. Health professionals and artificial intelligence applications can be used to help control the problem. Therefore, it is crucial that dermatologists use digital platforms more actively to share knowledge in the fields of cosmetology and dermatology by citing reliable references, making it easier for the general public to obtain correct information. We suggest that dermatologists should play a more active role on digital platforms by using understandable language, adhering to ethical values, and making references to cosmetic procedures, common diseases, and rare diseases.

## Conflicts of Interest

The authors declare no conflicts of interest.

## Supporting information


**Table S1.** Survey questions.

## Data Availability

The data that support the findings of this study are available on request from the corresponding author. The data are not publicly available due to privacy or ethical restrictions.
