# Supplementary material for: Dermatologists' Way of Informative Content About Dermatology and Cosmetology on Social Media
Source: J Cosmet Dermatol. 2025 Jul 21;24(7):e70148. doi: 10.1111/jocd.70148 (PMC12278336; doi:10.1111/jocd.70148)
Supplement: Supplementary file 1 — Table S1. Survey questions. [file JOCD-24-e70148-s001.docx]

Table S1. Survey questions

| **No** | **Question** |
| --- | --- |
| 1 | Sex   - Female - Male |
| 2 | Age: |
| 3 | Academic title   - Dermatology resident - Clinical dermatologist - Assistant professor - Associate professor - Professor |
| 4 | Years worked as a dermatologist (including residency training): |
| 5 | The institution you work for:   - Public hospital - Training and research hospital - University hospital - Private hospital - Private practice - Not currently working |
| 6 | Do you use social media?   - Yes - No (If the answer is no, the survey is over.) |
| 7 | Which applications do you use in social media?   - WhatsApp - Instagram - Facebook - YouTube - TikTok - Twitter (X) - Telegram - Linkedin - Others: |
| 8 | Do you actively use social media to share information on dermatology and cosmetology?   - Yes - No(If the answer is no, the survey is over.) |
| 9 | How do you choose the subjects for your social media content? By:   - Asking your followers - Prioritizing the diseases you encounter in daily practice - Considering the posts of other dermatologists - Considering the posts of non-dermatologist users such as influencers etc. - Considering the number of likes and interactions of the content - Other: |
| 10 | How do you share information about dermatology and cosmetology in your social media content?   - By making a video - By live streaming - Through question and answer - By sharing articles/articles - By sharing patient photo and diagnosis - Other: |
| 11 | What is your motivation for producing informative content in the field of dermatology and cosmetology on social media?   - Informing the public - Advertising - Visibility - Increasing the number of followers - Earning money (sponsorship etc.) - Interacting with other dermatologists - Contributing to the education of other dermatologists - Other |
| 12 | Do you think social media plays a role in patients' preference for dermatologists?   - Yes - No - I have no idea |
| 13 | Have you conducted or would you consider conducting scientific research on social media?   - Yes - No - I have no idea |
| 14 | Do you work with a social media consultant?   - Yes - No |
| 15 | Which subjects do you primarily share information about on social media?   - Daily skin/nail/hair care - Cosmetic procedures - Frequently encountered diseases - Relatively rare diseases - Skin cancers and skin cancer prevention - Other |
| 16 | If you share information about frequently encountered diseases, which subjects would you prefer to share about?   - Acne - Rosacea - Seborrheic dermatitis - Eczema - Hair loss - Psoriasis - Urticaria - Dry skin - Sun sensitivity - Viral diseases such as verruca etc. - Parasitic diseases - Fungal infections - Bacterial infections - Skin cancers - Others: |
| 17 | If you share information about rare dermatological diseases, which subjects would you prefer to share about?  … |
| 18 | Which cosmetic applications do you share information about?   - Botulinum toxin - Fillers - Threads - Energy-based devices - PRP/Mesotherapy - Lipolysis - I do not share cosmetic application content. |
| 19 | Do you share photos or videos of cosmetic procedures?   - Yes - No |
| 20 | Do you share photos or videos of surgical procedures?   - Yes - No |
| 21 | Do you get patient consent for photo or video content sharing?   - Yes - No |
| 22 | Do you provide treatment recommendations to your followers in your social media content?   - Yes - No |
| 23 | Do you recommend home remedies to your followers?   - Yes - No |
| 24 | Do you recommend dermocosmetic products to your followers?   - Yes - No |
| 25 | Do you recommend cosmetic procedures to your followers?   - Yes - No |
| 26 | Do you recommend medical treatment to your followers?   - Yes - No |
| 27 | Do you provide treatment and care recommendations for relatively rare diseases?   - Yes - No |
| 28 | Would you agree that institutional (through associations, etc.) rather than individual information sharing would be more effective?   - Yes, I agree - No, I don’t agree - I have no idea |
| 29 | Do you share the reference of the information that you are covering in the content?   - Yes - No |
| 30 | Which references do you use for the information you provide in the content?   - Literature data - Books - Google - Information brochures of associations - Youtube videos - Other: |
| 31 | Do you answer individual questions on social media?   - Yes - No |
| 32 | Do you share these questions in your content?   - Yes - No |
| 33 | How would you evaluate the language you use in the content you create?   - I use scientific/technical words. - I use words that dermatologists are familiar with. |
| 34 | Do you think that content shared on social media facilitates chronic disease management?   - Yes - No |
| 35 | Do you use a method to increase the visibility of posts?   - Yes, by sponsorship - Yes, by working to increase the number of likes - No - Other: |
| 36 | How much do you know about certain relevant legal regulations such as rules on advertising on social media and electronic media, patient privacy, personal and health data protection?   - Not at all - A few - Moderate - Very much |
